# Boron Reduced Copper Excess-Induced Oxidative Damage in *Citrus sinensis* by Modulating Reactive Oxygen Species and Methylglyoxal Formation and Their Detoxification Systems

**DOI:** 10.3390/antiox13030268

**Published:** 2024-02-22

**Authors:** Xu-Feng Chen, Huan-Huan Chen, Wei-Lin Huang, Wei-Tao Huang, Zeng-Rong Huang, Lin-Tong Yang, Xin Ye, Li-Song Chen

**Affiliations:** College of Resources and Environment, Fujian Agriculture and Forestry University, Fuzhou 350002, China; 2210807010@fafu.edu.cn (X.-F.C.); 2200807011@fafu.edu.cn (H.-H.C.); 2210807003@fafu.edu.cn (W.-L.H.); 2220807009@fafu.edu.cn (W.-T.H.); huangzengrong@fafu.edu.cn (Z.-R.H.); talstoy@fafu.edu.cn (L.-T.Y.); yexin1000@fafu.edu.cn (X.Y.)

**Keywords:** antioxidant enzymes, ascorbate-glutathione cycle, *Citrus sinensis*, glyoxalase, methylglyoxal detoxification system, reactive oxygen species, thiol-based antioxidant system

## Abstract

Citrus is mainly cultivated in acid soil with low boron (B) and high copper (Cu). In this study, *Citrus sinensis* seedlings were submitted to 0.5 (control) or 350 μM Cu (Cu excess or Cu exposure) and 2.5, 10, or 25 μM B for 24 weeks. Thereafter, H_2_O_2_ production rate (HPR), superoxide production rate (SAPR), malondialdehyde, methylglyoxal, and reactive oxygen species (ROS) and methylglyoxal detoxification systems were measured in leaves and roots in order to test the hypothesis that B addition mitigated Cu excess-induced oxidative damage in leaves and roots by reducing the Cu excess-induced formation and accumulation of ROS and MG and by counteracting the impairments of Cu excess on ROS and methylglyoxal detoxification systems. Cu and B treatments displayed an interactive influence on ROS and methylglyoxal formation and their detoxification systems. Cu excess increased the HPR, SAPR, methylglyoxal level, and malondialdehyde level by 10.9% (54.3%), 38.9% (31.4%), 50.3% (24.9%), and 312.4% (585.4%), respectively, in leaves (roots) of 2.5 μM B-treated seedlings, while it only increased the malondialdehyde level by 48.5% (97.8%) in leaves (roots) of 25 μM B-treated seedlings. Additionally, B addition counteracted the impairments of Cu excess on antioxidant enzymes, ascorbate-glutathione cycle, sulfur metabolism-related enzymes, sulfur-containing compounds, and methylglyoxal detoxification system, thereby protecting the leaves and roots of Cu-exposed seedlings against oxidative damage via the coordinated actions of ROS and methylglyoxal removal systems. Our findings corroborated the hypothesis that B addition alleviated Cu excess-induced oxidative damage in leaves and roots by decreasing the Cu excess-induced formation and accumulation of ROS and MG and by lessening the impairments of Cu excess on their detoxification systems. Further analysis indicated that the pathways involved in the B-induced amelioration of oxidative stress caused by Cu excess differed between leaves and roots.

## 1. Introduction

As with the other heavy metals (HMs), micronutrient copper (Cu) is also highly toxic for plants when it accumulates in large amounts in soil [1]. Cu is often used as a fungicide on fruit crops due to its high efficiency and low cost [2]. Cu-based fungicides have been widely and successively applied to control citrus fruit and foliar diseases [3]. The sustained and heavy application of Cu-based fungicides has led to the accumulation of Cu in orchard soil [4]. The accumulation and bioavailability of Cu in citrus orchard soil rise with the age of citrus trees [5]. In this scenario, excessive Cu accumulation in some old citrus orchard soils has become a common factor constraining citrus production due to the increased phytotoxicity of Cu and the slow transformation of Cu from active to inactive forms in soil [6].

A recent study from our research team demonstrated that boron (B) addition could counteract Cu exposure-induced reduction of growth via reducing root damage and Cu uptake and upgrading water and nutrition status in *Citrus sinensis* seedlings [7]. In another subsequent study, we investigated the effects of B addition on Cu-induced exudation of low molecular weight compounds (LMWCs; total free amino acids, total phenolics, total soluble sugars, malate, and citrate) by roots and their concentrations in leaves and roots, as well as related enzyme activities (gene expression levels) in leaves and roots of *C. sinensis* seedlings. Our results indicated that the B-mediated mitigation of Cu toxicity in *C. sinensis* seedlings involved enhanced internal detoxification of Cu by LMWCs in leaves and roots rather than increased exudation of LMWCs by roots [8]. So far, the mechanisms by which B mitigates Cu toxicity remains poorly understood.

It is known that plants have evolved various mechanisms to internally detoxify Cu [1,9,10]. Therefore, in addition to the detoxification of Cu by LMWCs, other Cu internal detoxification mechanisms may also play a role in the B-mediated reduction of citrus Cu toxicity. Plants exposed to excessive Cu often suffer from oxidative impairment and even cell death due to elevated formation and accumulation of reactive oxygen species (ROS) and methylglyoxal (MG) [11,12]. ROS can be detoxified by antioxidant enzymes (the first line of defense against oxidative stress) [13], ascorbate (ASC)-glutathione cycle [14], and thiol-based antioxidant system (the second line of defense against oxidative stress) [13]. MG degradation is mainly carried out by glyoxalase (Gly) system [15]. It has been pointed out that the coordinated actions of ROS and MG scavenging systems play an important role in addressing the elevated formation of ROS and MG [16]. So far, some researchers have examined the changes in ROS and MG scavenging systems caused by Cu excess and their role in Cu tolerance of plants [4,12,17,18]. Yet, the roles of ROS and MG formation and their scavenging systems in the B-mediated tolerance to oxidative damage caused by Cu excess remains to be revealed. Researches demonstrated that ASC-, reduced glutathione (GSH)-, NO-, acetylsalicylic acid-, or melatonin-mediated tolerance to Cu excess in mungbean (*Vigna radiata*) [19], cucumber (*Cucumis sativus*) [20], and rice (*Oryza sativa*) [21], and elevated pH-mediated tolerance to Cu excess in *C. sinensis* [12] involved reduced oxidative damage by reducing Cu-stimulated formation of ROS and MG and by counteracting the impairments of Cu excess on ROS (antioxidant enzymes, ASC-glutathione cycle, and thiol-based antioxidant system) and MG detoxification systems. Therefore, the B-mediated tolerance to oxidative damage caused by Cu excess in plants might be explained in this way.

Citrus is mainly cultivated in acid soil having low B and high Cu [22,23]. For the first time, we used *C. sinensis* seedlings as study objects to investigate the effects of B addition on ROS and MG formation, and ROS and MG detoxification systems in the leaves and roots of Cu-exposed seedlings. The study aimed to corroborate the hypothesis that B addition mitigated Cu excess-induced oxidative damage in leaves and roots by reducing Cu excess-induced formation and accumulation of ROS and MG and by counteracting the impairments of Cu excess on ROS (antioxidant enzymes, ASC-glutathione cycle, and thiol-based antioxidant system) and MG detoxification systems. This study was conducted on the basis of previous studies [7,8] and could complement the previous studies, since it might provide some novel information on the B-mediated internal detoxification mechanisms of Cu.

## 2. Materials and Methods

### 2.1. Seedling Culture and Treatments

‘Xuegan’ (*Citrus sinensis* (L.) Osbeck) seeds were collected from Minan village, Tingjiang town, Mawei district, Fuzhou, China. Seedling culture and treatments used in this experiment were based on our recent research [7]. Six weeks after ‘Xuegan’ seed germination, uniform seedlings were transplanted to 6 L flowerpot (2 seedlings pot^−1^) filled with sand, and grown in a greenhouse under natural conditions at Fujian Agriculture and Forestry University, Fuzhou, China (26°5′ N, 119°14′ E). Seven weeks after transplantation, each pot was supplied 6 times weekly with freshly prepared nutrition solution (adjusted to pH 4.8 with HCl) at a Cu level of 350 (Cu350, Cu excess or Cu exposure) or 0.5 (Cu0.5) μM from CuCl_2_ and a B level of 25 (B25), 10 (B10), or 2.5 (B2.5) μM from H_3_BO_3_ until dripping. There were 15 flowerpots per treatment in a completely randomized design. After 24 weeks of Cu and B treatments, ~0.5 cm in length white root tips and 0.6 cm-diameter discs from the recent fully expanded (~7-week-old) leaves from 15 seedlings (1 seedling per flowerpot) were collected at noon and immersed immediately into liquid nitrogen, then stored at −80 °C freezer until the extraction of metabolites and enzymes.

### 2.2. Assays of HPR, SAPR, and Metabolites in Roots and Leaves

H_2_O_2_ production rate (HPR) and superoxide anion (O_2_^.-^) production rate (SAPR) were assayed using fresh root tips and leaf discs harvested from the seedlings without sampling. HPR and SAPR were assayed by the reduction of nitroblue tetrazolium (NBT) and the oxidation of guaiacol, respectively [24]. Malondialdehyde (MDA) was estimated using the modified thiobarbituric acid-reactive-substances after extraction with 80% (*v*/*v*) ethanol [25]. MG was quantified using N-acetyl-L-cysteine assay after extraction with 5% (*w*/*v*) HClO_4_ [26].

Oxidized glutathione (GSSG), GSH, ASC, and dehydroascorbate (DHA) were determined spectrophotometrically by a method of enzymatic analysis [24].

Metallothioneins (MTs) were determined spectrophotometrically with 5,5-dithiobis-2-nitrobenzoic acid (DTNB) [27] after samples were extracted with 20 mM TRIS-HCl (pH 8.6) containing 0.5 M sucrose and 0.01% β-mercaptoethanol. GSH and total non-protein thiols (TNP-SH) were determined spectrophotometrically with DTNB after samples were extracted with 5% (*v*/*v*) sulphosalicylic acid. The calculation formula for phytochelatins (PCs) was: PCs = TNP-SH − GSH [28]. 

### 2.3. Assays of Enzyme Activities in Leaves and Roots

#### 2.3.1. Antioxidant Enzymes

Approximately 30 mg frozen samples were extracted with 2 mL of 50 mM KH_2_PO_4_-KOH (pH 7.5) containing 0.5% Triton X-100, 1 mM EDTA, and 5% insoluble polyvinylpolypyrrolidone (PVPP). The extracts were then centrifuged at 4 °C and 13,000 *g* for 10 min, and the supernatants were used immediately for the measurements of superoxide dismutase (SOD), guaiacol peroxidase (GuPX), catalase (CAT), glutathione reductase (GR), monodehydroascorbate (MDHA) reductase (MDHAR), DHA reductase (DHAR), and ASC peroxidase (APX) activities [12]. SOD activity was determined at 560 nm in a reaction mixture containing methionine, NBT, riboflavin, and enzyme extract [29]. GuPX activity was determined spectrophotometrically via following the increase of absorbance at 470 nm in a mixture (1 mL) of 100 mM potassium phosphate (pH 6.0), 16 mM guaiacol, 5 μL 10% (*w*/*v*) H_2_O_2_, and 20 μL enzyme extract [12]. CAT activity was measured at 240 nm via following the decline in absorbance in a mixture (1 mL) of 100 mM potassium phosphate buffer (pH 6.0), 10 μL enzyme extract, and 10 μL 10% (*w*/*v*) H_2_O_2_ [12]. GR activity was determined at 340 nm following a decline in absorbance in a mixture (1 mL) of 100 mM TRIS-HCl (pH 8.0), 1 mM EDTA, 1 mM GSSG, 100 μL enzyme extract, and 0.2 mM NADPH [12]. APX activity was determined at 290 nm by following the decline of absorbance in a mixture (1 mL) of 50 mM HEPES-KOH (pH 7.6), 0.5 mM ASC, 0.1 mM EDTA, 50 μL enzyme extract, and 0.2 mM H_2_O_2_ [12]. MDHAR activity was assayed via following the decline of absorbance at 340 nm in a mixture (1 mL) of 50 mM HEPES-KOH (pH 7.6), 0.1 mM NADH, 2.5 mM ASC, 20 μL enzyme extract, and 0.25 U of ASC oxidase [12]. DHAR activity was measured via following the rise of absorbance at 265 nm in a mixture (1 mL) of 100 mM HEPES-KOH (pH 7.0), 2.5 mM GSH, 0.1 mM EDTA, 100 μL enzyme extract, and 0.2 mM DHA [12].

#### 2.3.2. Sulfur Metabolism-Related Enzymes and Glyoxalases

Approximately 30 mg frozen samples were extracted with 1 mL of 100 mM TRIS-HCl (pH 8.0) containing 2 mM dithiothreitol (DTT), 10 mM EDTA, and 4% (*w*/*v*) insoluble PVPP. The extracts were then extracted at 4 °C and 13,000 *g* for 10 min at 4 °C. The resultants supernatants were used immediately for the assays of ATP sulfurylase (ATPS), γ-glutamylcysteine synthetase (γGCS), adenosine 5′-phosphosulphate (APS) reductase (APR), cysteine (Cys) synthase (CS), sulfite reductase (SiR), γ-glutamyltransferase (γGT), Gly I, and Gly II activities [12]. γGCS was determined in a mixture (1 mM) of 100 mM TRIS-HCl (pH 8.0), 2 mM EDTA, 150 mM KCl, 20 mM MgCl_2_, 0.2 mM NADH, 5 mM ATP, 10 mM α-aminobutyrate, 2 mM phosphoenolpyruvate (PEP), 10 mM glutamate, 10 U of lactate dehydrogenase (LDH), 7 U of pyruvate kinase (PK), and 100 μL enzyme extract [12]. γGT was measured according to Zhang et al. [12]. Enzyme extract (100 μL) was incubated at 30 °C for 30 min with 100 mM TRIS-HCl (pH 8.0), 2.5 mM L-γ-glutamyl-p-nitroanilide, and 20 mM glycylglycine (Gly-Gly) in a total volume of 1 mL, then the reaction was terminated by adding 1 mL of 25% (*w*/*v*) trichloroacetic acid (TCA). The resultant p-nitroaniline (ε = 1.74 mM^−1^ cm^−1^) was measured at 405 nm. SiR was measured in a mixture (1 mL) of 10 mM TRIS-HCl (pH 7.5), 0.5 mM Na_2_SO_3_, 0.1 mM EDTA, 0.2 mM NADPH, and 100 μL enzyme extract [12]. CS activity was assayed according to Warrilow and Hawkesford [30]. Enzyme extract (20 μL) was incubated at 25 °C for 10 min with 0.2 M TRIS-HCl (pH7.5), 3 mM Na_2_S, 10 mM DTT, 20 μL extract, and 5 mM O-acetyl-L-serine (OAS) in a total volume of 0.8 mL. This reaction was started and terminated by adding OAS and 0.2 mL of 1.5 M TCA, respectively. The resultant cysteine was determined by ninhydrin method. APR activity was determined according to Trüper and Rogers [31] with some modification. One milliliter of mixture for ferricyanide-coupled assay contained 50 mM TRIS-HCl (pH 8.0), 8 mM EDTA, 4 mM Na_2_SO_3_, 0.5 mM K_3_Fe(CN)_6_, 0.4 mM AMP, and 100 μL enzyme extract. The decrease in absorbance was recorded at 420 nm and 25 °C against a blank containing the mixture without enzyme extract. ATPS activity was assayed according to Zhang et al. [12]. Enzyme extract (100 μL) was incubated for 15 min at 37 °C with 80 mM TRIS-HCl (pH 8.0), 7 mM MgCl_2_, 2 mM Na_2_ATP, 5 mM Na_2_MoO_4_, and 0.032 U mL^−1^ of sulfate-free inorganic pyrophosphatase in a total volume of 0.6 mL. The reaction was started and stopped by adding enzyme extract and 2 mL of 20% (*w*/*v*) TCA, respectively. The resultant phosphate was determined according to Ames [32]. The blank contained the same mixture and enzyme extract without Na_2_MoO_4_. Gly I activity was measured in a reaction mixture (1 mM) of 100 mM potassium phosphate buffer (pH 7.0), 1.7 mM GSH, 15 mM MgSO_4_, 100 μL enzyme extract, and 3.5 mM MG. The reaction was initiated by adding MG. The rise in absorbance was recorded at 25 °C and 240 nm (extinction coefficient of 3.37 mM^−1^ cm^−1^) against a blank containing the reaction mixture without enzyme extract [33]. Gly II activity was assayed by monitoring the formation of GSH at 25 °C and 412 nm (extinction coefficient of 13.6 mM^−1^ cm^−1^) in a reaction mixture (1 mL) of 100 mM TRIS-HCl (pH 7.2), 0.2 mM DTNB, 100 μL enzyme extract, and 1 mM S-D-lactoylglutathione (SLG) [33]. The extraction of glutathione S-transferase (GST) was the same as the extraction of antioxidant enzymes mentioned above. GST activity was determined via following the rise of absorbance at 340 nm in a mixture (1 mL) of 100 mM TRIS-HCl (pH6.5), 1.5 mM GSH, 50 μL enzyme extract, and 1 mM 1-chloro-2,4-dinitrobenzene (CDNB). The reaction was initiated by adding CDNB [34].

### 2.4. Data Analysis

Principal coordinate analysis (PCoA) was implemented by ChiPlot (https://www.chiplot.online/, accessed on 15 July 2023). Principal component analysis (PCA) and Pearson correlation coefficients were calculated using SPSS statistical software (version 17.0, IBM, NY, USA). Data were examined by two-way ANOVA followed by the least significant difference (LSD) at *p* < 0.05 using DPS 7.05 (Hangzhou RuiFeng Information Technology Co., Ltd., Hangzhou, China).

## 3. Results

### 3.1. Effects of Cu and B Treatments on HPR, SAPR, MG, and MDA in Leaves and Roots

**Figure 1:**
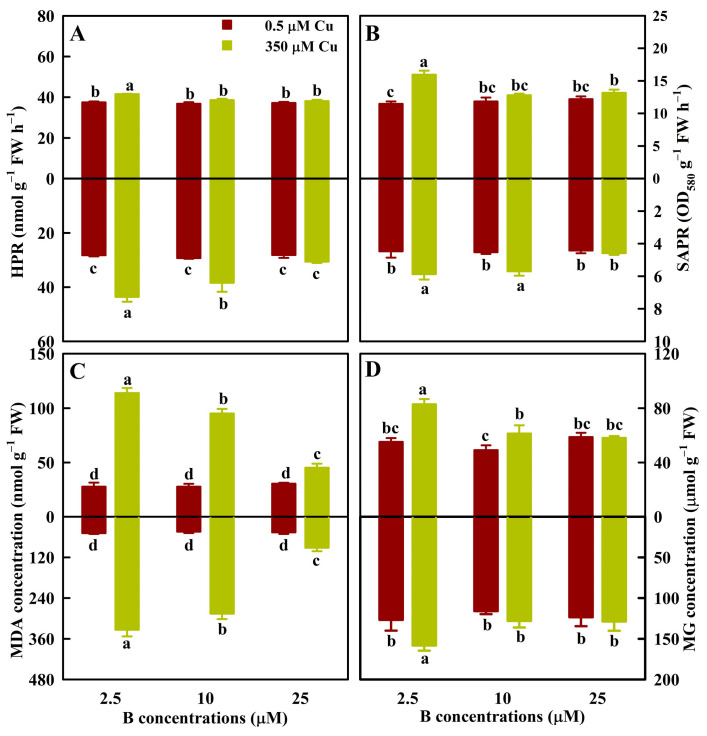
The mean (± SE, *n* = 4) HPR (**A**), SAPR (**B**), and concentrations of MDA (**C**) and MG (**D**) in leaves (above column) and roots (below column) of *C. sinensis* seedlings under different Cu and B treatments. The means followed by the different letters were significantly different according to two-way ANOVA followed by the LSD at *p* < 0.05. The *F* values and the *p* values for Cu, B, and Cu × B were listed in Table S1.

For leaves, Cu350 increased HPR (SAPR) by 10.9% (38.9%) at B2.5, MDA concentrations by 312.4%, 244.3%, and 48.5%, respectively, at B2.5, B10, and B25, and MG concentrations by 50.3% and 25.1%, respectively, at B2.5 and B10. Cu350 did not alter HPR, SAPR, and MG concentrations at the other B levels. At Cu350, HPR, SAPR, and MG concentrations were higher at B2.5 than at B10 and B25, and MDA concentration increased with decreasing B (Figure 1).

For roots, Cu350 increased HPR (SAPR) by 54.3% (31.4%) and 31.0% (26%), respectively, at B2.5 and B10, MDA concentrations by 585.4%, 543.5%, and 97.8%, respectively, at B2.5, B10, and B25, and MG concentration by 24.9% at B2.5. Cu350 did not affect HPR, SAPR, and MG levels at the other B levels. At Cu350, HPR and MDA concentrations increased with the decrease of B supply, SAPR was higher at B2.5 and B10 than at B25, and MDA concentration was higher at B2.5 than at B10 and B25 (Figure 1).

B supply did not significantly alter the four indexes in leaves of 0.5 μM Cu-treated seedlings (LCu0.5) and roots of 0.5 μM Cu-treated seedlings (RCu0.5; Figure 1).

### 3.2. Effects of Cu and B Treatments on ROS and MG Scavenging Enzyme Activities in Leaves and Roots

**Figure 2:**
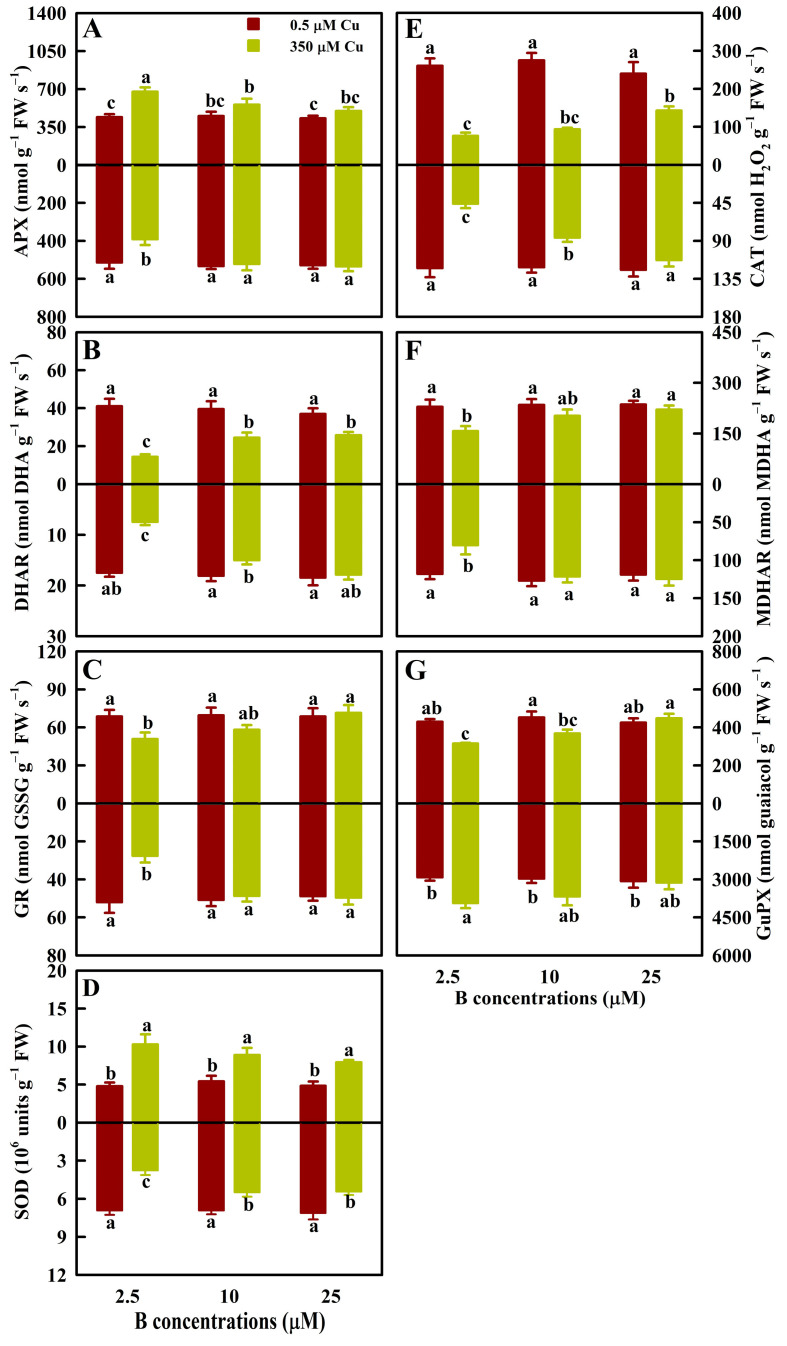
The mean (± SE, *n* = 4) activities of APX (**A**), DHAR (**B**), GR (**C**), SOD (**D**), CAT (**E**), MDHAR (**F**), and GuPX (**G**) in leaves (above column) and roots (below column) of *C. sinensis* seedlings under different Cu and B treatments. The means followed by the different letters were significantly different according to two-way ANOVA followed by the LSD at *p* < 0.05. The *F* values and the *p* values for Cu, B, and Cu × B were listed in Table S1.

**Figure 3:**
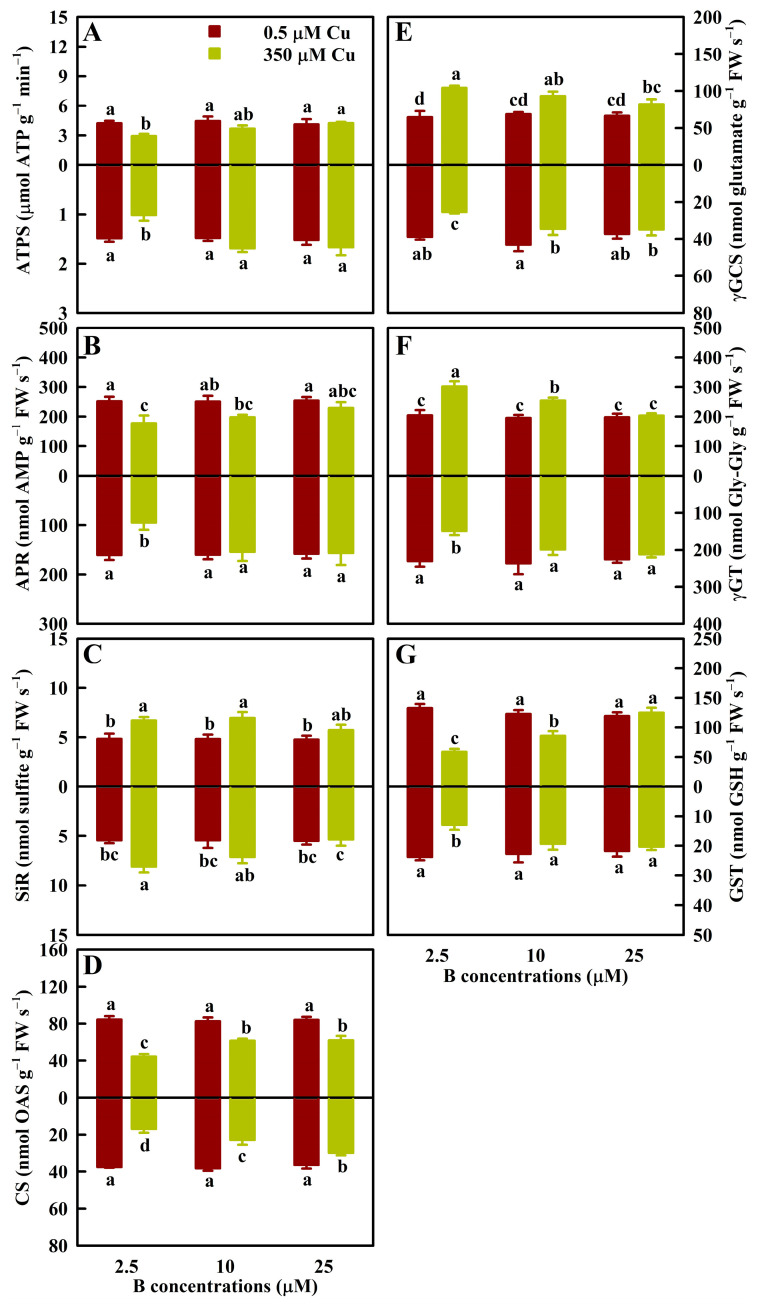
The mean (± SE, *n* = 4) activities of ATPS (**A**), APR (**B**), SiR (**C**), CS (**D**), γGCS (**E**), γGT (**F**), and GST (**G**) in leaves (above column) and roots (below column) of *C. sinensis* seedlings under different Cu and B treatments. OAS, O-acetylserine; Gly-Gly, glycylglycine. The means followed by the different letters were significantly different according to two-way ANOVA followed by the LSD at *p* < 0.05. The *F* values and the *p* values for Cu, B, and Cu × B were listed in Table S1.

To reveal the mechanisms underlying the B-mediated alleviation of oxidative damage in leaves of 350 μM Cu-treated seedlings (LCu350) and roots of 350 μM Cu-treated seedlings (RCu350), we examined the impacts of Cu and B treatments on the activities of antioxidant enzymes (Figure 2), sulfur (S) metabolism-related enzymes (Figure 3), and glyoxalases (Table 1) in leaves and roots. As shown in Figure 2, Cu350 increased and decreased the APX activities in leaves of 2.5 μM B-treated seedlings (LB2.5) and roots of 2.5 μM B-treated seedlings (RB2.5), respectively, and decreased the MDHAR and GR activities in LB2.5 and RB2.5, but it did not significantly alter the APX, MDHAR, and GR activities in leaves and roots of 10 and 25 μM B-treated seedlings. Cu350 decreased the CAT and DHAR activities in leaves and their activities in RB2.5 and roots of 10 μM B-treated seedlings (RB10), but Cu350 did not significantly alter their activities in roots of 25 μM B-treated seedlings (RB25). Cu350 increased and decreased the SOD activities in leaves and roots, respectively. Cu350 reduced the GuPX activities in LB2.5 and leaves of 10 μM B-treated seedlings (LB10) and increased the GuPX activity in RB2.5, but Cu350 did not significantly alter the GuPX activities in RB10, leaves of 25 μM B-treated seedlings (LB25), and RB25. Overall, the impacts of Cu350 on the antioxidant enzyme activities were greater in LB2.5 and RB2.5 than in LB25 and RB25.

B25 decreased (increased) the APX activity in LCu350 (RCu350), increased the CAT, DHAR, MDHAR, and GR activities in LCu350 and RCu350 and the SOD (GuPX) activity in RCu350 (LCu350), and did not affect the SOD (GuPX) activity in LCu350 (RCu350) relative to B2.5. B supply did not affect their activities in LCu0.5 and RCu0.5 (Figure 2).

As shown in Figure 3, Cu350 reduced the ATPS, APR, and GST activities in LB2.5 and RB2.5, but not in LB10, RB10, LB25, and RB25 except a decreased GST activity in LB10. Cu350 increased (decreased) the γGCS and γGT activities in LB2.5 and LB10 (RB2.5 and RB10) except an unaltered γGT activity in RB10, but not in LB25 and RB25. Cu350 increased the SiR activities in LB2.5, LB10, and RB2.5, but not in LB25, RB10, and RB25. Cu350 lowered the CS activities less in LB25 and RB25 than in LB2.5 and RB2.5.

B25 increased the ATPS, APR, CS, and GST activities in LCu350 and RCu350 and the γGCS and γGT activities in RCu350, reduced the γGCS and γGT activities in LCu350 and the SiR activity in RCu350, and did not alter the SiR activity in LCu350 relative to B2.5. B supply had no effect on their activities in LCu0.5 and RCu0.5 (Figure 3).

Cu350 decreased the Gly I activities in LB2.5 and RB2.5 and the Gly II activity in RB2.5, but it improved the Gly II activity in LB2.5. Cu350 had no effect on their activities in RB10, RB25, LB10, and LB25 (Table 1). B25 increased the Gly I activities in LCu350 and RCu350 and the Gly II activity in RCu350, but it had no effect on the Gly II activity in LCu350 relative to B2.5. B supply did not affect their activities in LCu0.5 and RCu0.5 (Table 1).

### 3.3. Effects of Cu and B Treatments on Antioxidant and S-Containing Compound Levels in Leaves and Roots

**Figure 4:**
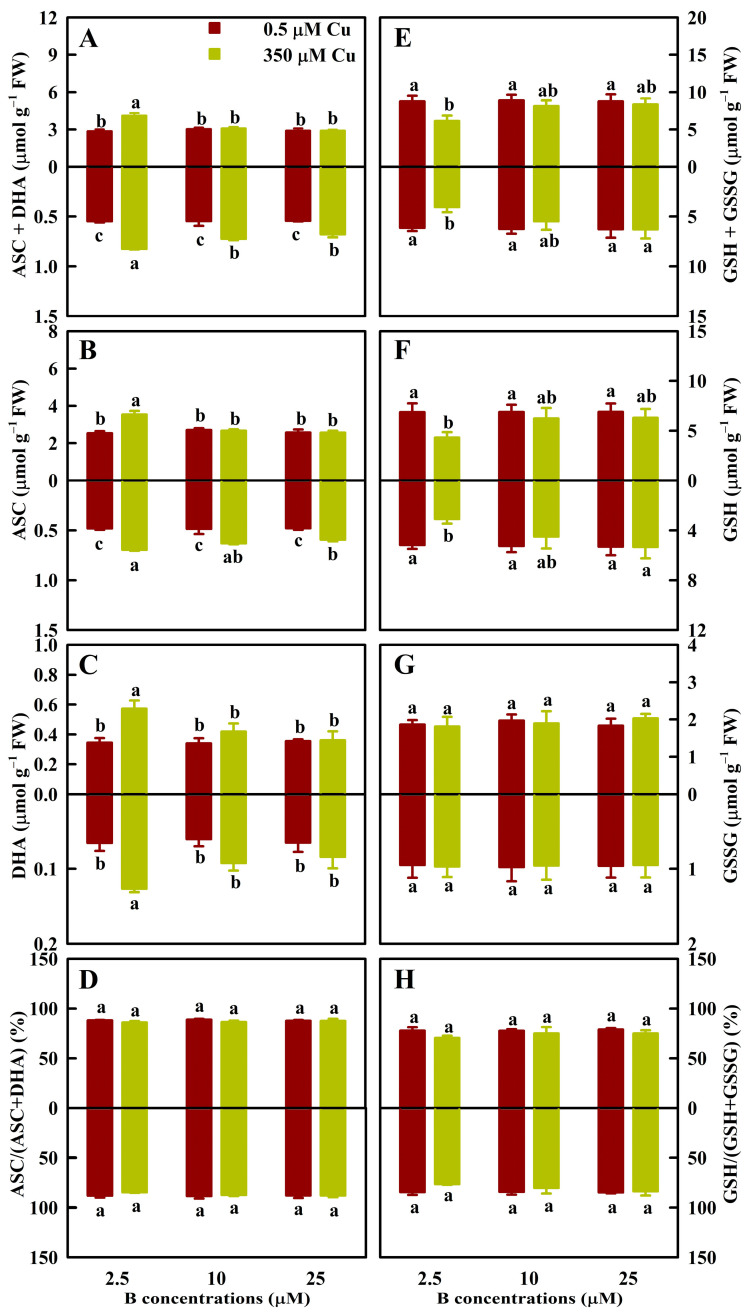
The mean (± SE, *n* = 4) ASC + DHA (TA; (**A**)), ASC (**B**), and DHA (**C**) concentrations, ASC/TA ratio (**D**), GSH + GSSG (TG; (**E**)), GSH (**F**), and GSSG (**G**) concentrations, and GSH/TG ratio (**H**) in leaves (above column) and roots (below column) of *C. sinensis* seedlings under different Cu and B treatments. The means followed by the different letters were significantly different according to two-way ANOVA followed by the LSD at *p* < 0.05. The *F* values and the *p* values for Cu, B, and Cu × B were listed in Table S1.

**Figure 5:**
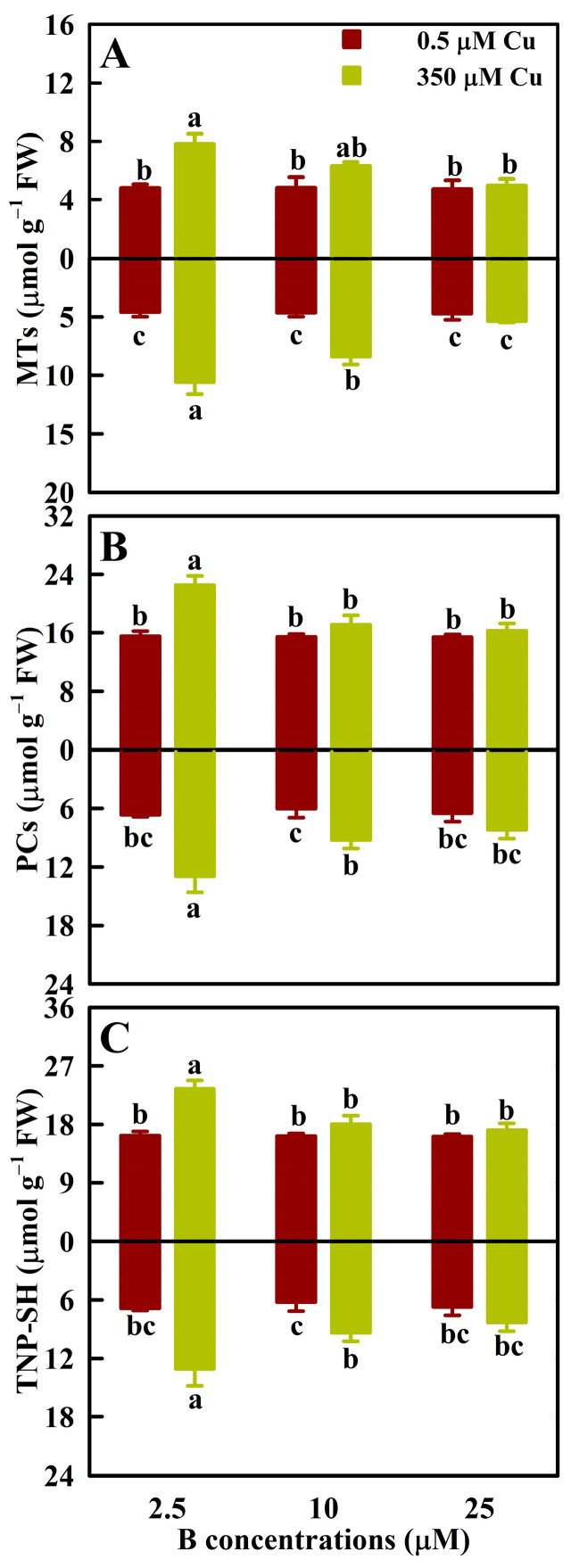
The mean (±SE, *n* = 4) concentrations of MTs (**A**), PCs (**B**), and TNP-SH (**C**) in leaves (above column) and roots (below column) of *C. sinensis* seedlings under different Cu and B treatments. The means followed by the different letters were significantly different according to two-way ANOVA followed by the LSD at *p* < 0.05. The *F* values and the *p* values for Cu, B, and Cu × B were listed in Table S1.

We assayed the concentrations of antioxidants (Figure 4) and S-containing compounds (Figure 5) in leaves and roots. Cu350 increased the TA concentrations by 43.0%, 50.8%, 32.5%, and 25.1% in LB2.5, RB2.5, RB10, and RB25, respectively, the ASC concentrations by 39.9%, 44.8%, 30.0%, and 24.5% in LB2.5, RB2.5, RB10, and RB25, respectively, and the DHA concentrations by 65.8% and 94.6% in LB2.5 and RB2.5%, respectively. However, Cu350 did not change the TA, ASC, and DHA concentrations in LB10 and LB25, and the DHA concentrations in RB10 and RB25. Cu350 reduced the TG and GSH levels in LB2.5 (RB2.5) by 29.9% (33.9%) and 37.2% (40.5%), respectively, but it did not alter their levels in LB10 and LB25 (RB10 and RB25) (Figure 4). B25 elevated the TA, ASC, and DHA levels, but it reduced the TG and GSH levels in LCu350 and RCu350 relative to B2.5. B supply did not affect their levels in LCu0.5 and RCu0.5. Cu and B treatments did not alter GSSG concentrations and ASC/TA and GSH/TG ratios in leaves and roots (Figure 4). 

As shown in Figure 5, Cu350 increased the levels of MTs, PCs, and TNP-SH by 62.1%, 43.7%, and 45.4% in LB2.5, respectively, and by 131.2% (79.9%), 91.5% (50.9%), and 93.8% (52.3%) in RB2.5 (RB10), respectively. However, Cu350 did not affect their concentrations in LB10, LB25, and RB25. B25 reduced the levels of PCs, MTs, and TNP-SH in LCu350 and RCu350, but not in LCu0.5 and RCu0.5 relative to B2.5.

### 3.4. PCoA, PCA, and Regression Analysis for All the 31 Indexes in Leaves and Roots

**Figure 6:**
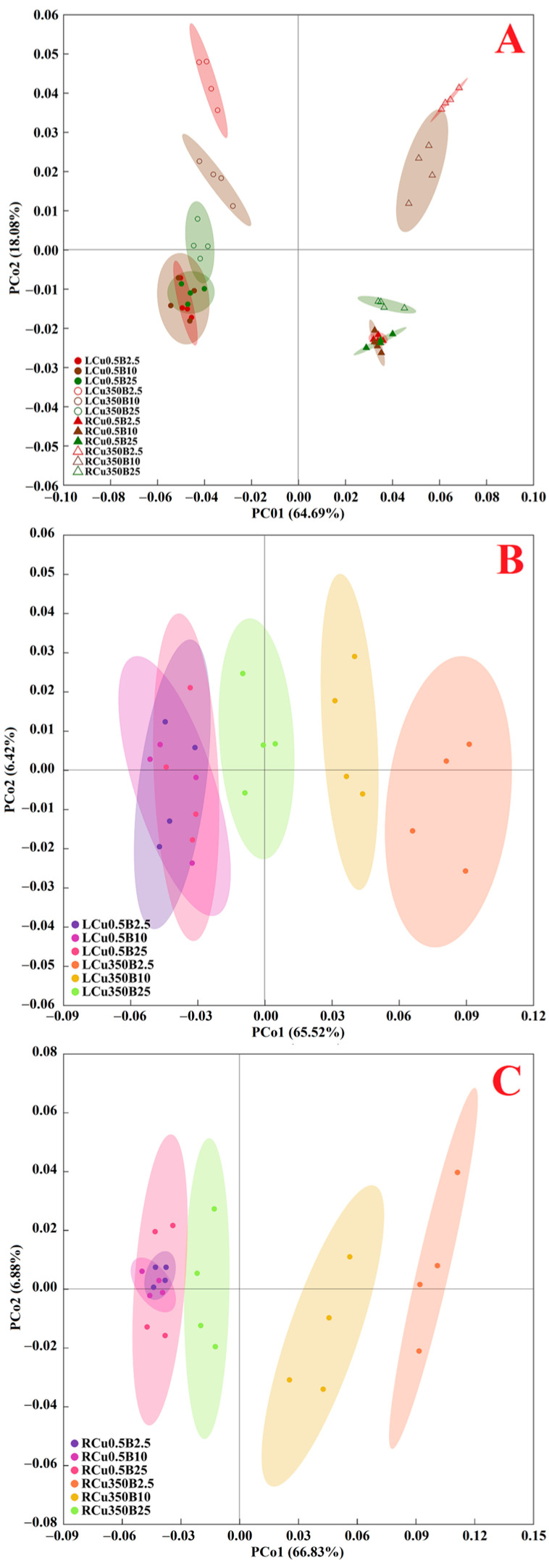
PCoA plots of 31 indexes for both leaves and roots (**A**), leaves (**B**), and roots (**C**) from *C. sinensis* seedlings treated with 0.5 or 350 µM Cu and 2.5, 10, or 25 µM B. RCu0.5B2.5, roots of 0.5 μM Cu + 2.5 μM B-treated seedlings; RCu0.5B10, roots of 0.5 μM Cu + 10 μM B-treated seedlings; RCu0.5B25, roots of 0.5 μM Cu + 25 μM B-treated seedlings; RCu350B2.5, roots of 350 μM Cu + 2.5 μM B-treated seedlings; RCu350B10, roots of 350 μM Cu + 10 μM B-treated seedlings; RCu350B25, roots of 350 μM Cu + 25 μM B-treated seedlings; LCu0.5B2.5, leaves of 0.5 μM Cu + 2.5 μM B-treated seedlings; LCu0.5B10, leaves of 0.5 μM Cu + 10 μM B-treated seedlings; LCu0.5B25, leaves of 0.5 μM Cu + 25 μM B-treated seedlings; LCu350B2.5, leaves of 350 μM Cu + 2.5 μM B-treated seedlings; LCu350B10, leaves of 350 μM Cu + 10 μM B-treated seedlings; and LCu350B25, leaves of 350 μM Cu + 25 μM B-treated seedlings.

To understand the response patterns of ROS and MG generation and their removal systems in both leaves and roots to Cu and B treatments, a PCoA was conducted using the 31 indexes, including HPR, SAPR, MG, MDA, 16 enzymes, eight antioxidants and ratios, and three S-containing compounds (Figure 6A). PCo1 separated the impacts of leaves and roots. PCo2 separated the effects of Cu excess in leaves and roots.

Also, a PCoA was carried out using the above 31 indexes to characterize leaf or root response patterns to Cu and B treatments (Figure 6B,C). Our results indicated that in both leaves and roots, the three treatments with 0.5 μM Cu (i.e., Cu0.5B2.5, Cu0.5B10, and Cu0.5B25) were highly clustered in the left side, but the three treatments with 350 μM Cu (i.e., Cu350B2.5, Cu350B10, and Cu350B25) were not clustered together. This implied that PCo1 could separate the impacts of Cu excess, and identify the variations in the impacts of exogenous B application. In the PCoA plots, we observed that the distance between LCu350 (RCu350) and LCu0.5 (RCu0.5) reduced with elevating B supply, suggesting that B addition counteracted Cu excess-induced changes in ROS and MG formation and their scavenging systems.

Additionally, a PCA was made using 62 indexes (31 leaf + 31 root indexes) to understand the response patterns of leaves and roots to Cu excess at B2.5, B10, or B25 (Figure S1A–C), and to B at Cu0.5 or Cu350 (Figure S1D,E). The contributions of PC1 and PC2 to the total variation decreased from 79.8% (71.2% for PC1 and 8.6% for PC2 at B) at B2.5 to 45.9% (24.0% for PC1 and 21.9% for PC2) at B25, and the contributions of PC1 and PC2 to the total variation were greater at Cu350 than at Cu0.5.

We conducted a regression analysis using all of the 31 indexes in leaves and/or roots (Figure S2). For leaves, a significant and positive relationship existed between any two indexes of HPR, SAPR, MG, and MDA. MG and MDA were significantly and positively related to APX, SOD, SiR, γGT, γGCS, Gly II, TA, ASC, DHA, MTs, PCs, and TNP-SH, and negatively related to GuPX, MDHAR, DHAR, CAT, GR, CS, GST, ATPS, APR, ASC/TA, TG, GSH, and GSH/TG with a few of exceptions (Figure S2A).

For roots, a significant and positive relationship existed between any two indexes of SAPR, HPR, MG, and MDA except the correlations between MG and SAPR (*r* = 0.744) and between MDA and MG (*r* = 0.781). MG and MDA were significantly and positively correlated to GuPX, SiR, TA, ASC, DHA, MTs, PCs, and TNP-SH, and negatively related to SOD, GR, CAT, APX, MDHAR, DHAR, γGCS, γGT, GST, APR, CS, Gly I, Gly II, ASC/TA, TG, GSH and GSH/TG with a few of exceptions (Figure S2B).

There were 18 and 6 indexes in the leaves that were significantly positively and negatively correlated with the corresponding indexes in the roots (Figure S2C).

## 4. Discussion

### 4.1. Cu and B Treatments Exhibited an Interactive Influence on ROS and MG Formation and Their Detoxification Systems in Leaves and Roots

On the basis of previous studies [7,8], we further investigated the effects of Cu and B treatments on 62 indexes in leaves (31 indexes) and roots (31 indexes). We found that at B2.5, B10, and B25, Cu350 altered 56 (28 leaf + 28 root), 24 (11 leaf + 13 root), and 10 (5 leaf + 5 root) indexes, respectively (Figure 1, Figure 2, Figure 3, Figure 4 and Figure 5 and Table 1). Obviously, the impacts of Cu350 on the 62 indexes reduced with increasing B supply. This was supported by the PCoA (Figure 6B,C) and PCA (Figure S1A–C), and the findings that B addition mitigated the effects of Al excess on ROS generation and removal in *Poncirus trifoliata* roots [35] and leaves [36], and the impacts of cadmium (Cd) excess on ROS production and removal in wheat roots and shoots [37]. A study from our laboratory showed that Cu levels in LCu350 and RCu350 decreased with elevating B supply [7]. This could explain why the changes of the 62 indexes caused by Cu350 decreased with increasing B supply. Compared with B25, B2.5 and B10 altered 49 (22 leaf + 27 root) and 9 (3 leaf + 6 root) parameters in LCu350 and RCu350, respectively, while B supply did not alter these parameters in LCu0.5 and RCu0.5 (Figure 1, Figure 2, Figure 3, Figure 4 and Figure 5 and Table 1), indicating that B supply altered ROS and MG formation and their scavenging systems in RCu350 and LCu350, but not in RCu0.5 and LCu0.5. This was also supported by the PCA (Figure S1D,E). As shown in Table S1, Cu and B treatments had significant interactive impacts on 25 (15 leaf + 10 root) out of 62 parameters. To sum up, Cu and B treatments had an interactive influence on ROS and MG generation and their detoxification systems in leaves and roots. This agreed with our previous results that Cu and B treatments had an interactive impact on *C. sinensis* growth [7].

### 4.2. B Addition Protected the Leaves and Roots of Cu-Exposed Seedlings from Oxidative Injury via the Coordinated Actions of ROS and MG Scavenging Systems

Regression analysis indicated that MDA concentrations in leaves and roots was significantly and positively related to SAPR, HPR, and MG (Figure S2A,B). This demonstrated that Cu350 elevated the biosynthesis and accumulation of ROS and MG, which led to oxidative damage in leaves and roots (Figure 1). Similar results have been observed in the leaves (shoots) and roots of Cu-exposed citrus [12,17], *Malus prunifolia* [4], and *Brassica rapa* [38] plants.

Evidence shows that B addition can mitigate metals-induced oxidative damage by regulating antioxidant enzyme activities and ASC-glutathione cycle, and reducing ROS production [35,36,39,40]. As expected, B addition reduced the ROS and MG generation and accumulation in RCu350 and LCu350, and mitigated the impairments of Cu excess on ROS and MG scavenging systems in leaves and roots (Figure 1, Figure 2, Figure 3, Figure 4 and Figure 5 and Table 1). Antioxidant enzymes form the first line of defense against oxidative stress. Among them, SOD and CAT mutually serve as vital enzymes in ROS detoxification [13]. O_2_^.-^ is often the first ROS formed in plant cells [41]. SOD is the first line of defense against ROS, since it can dismutate O_2_^.-^ into H_2_O_2_ and O_2_. The H_2_O_2_ formed is then converted into H_2_O and O_2_ by CAT in peroxisomes and mitochondria [42]. Additionally, peroxidases play a key role in the removal of H_2_O_2_ in plants through its various cellular types [43]. We observed that B addition mitigated Cu excess-induced decreases of CAT activities in leaves and roots, and SOD (GuPX) activity in roots (in leaves), and increases of SOD (GuPX) activity in leaves (roots) (Figure 2). This agreed with the findings that B addition mitigated Cd exposure-induced decreases of SOD and GuPX activities in leaves and roots, and CAT activity in roots, and increase of CAT activity in leaves of rice seedlings [39].

ASC-glutathione plays a central role in preventing oxidative damage to plant cells, and is mainly composed of four antioxidant enzymes (DHAR, MDHAR, APX, and GR) and two antioxidants (GSH and ASC) [14]. APX, which utilizes ASC to reduce H_2_O_2_ to H_2_O, with the concomitant production of MDHA, is one of the key enzymes in the cycle [43]. MDHA can be reduced to ASC by MDHAR. DHAR can reduce DHA back to ASC by using two GSH molecules, while producing a GSSG molecule. Using NAD(P)H as the substrate, GR can convert GSSG back to GSH [44]. ASC and GSH are the two most prevalent soluble antioxidants in plant cells [43]. GSH and ASC can also chelate metal ions, thereby scavenging them and lowering their catalytic activity to form ROS [45]. Tahjib-Ul-Arif et al. demonstrated that GSH- and/or ASC-mediated amelioration of rice Cu excess involved decreased level of Cu in shoots, and increased activities of CAT, GuPX, and APX and reduced accumulation of H_2_O_2_ and MDA in leaves [18]. Our results suggested that B addition alleviated the impairments of Cu excess on ASC-glutathione cycle in leaves and roots (Figure 2 and Figure 4).

Thiol-based antioxidant system constitutes the second line of defense against oxidative stress [13]. S-containing compounds (GSH, PCs, and MTs) are biosynthesized by ATPS and other S metabolism-related enzymes and play a vital role in plant stress tolerance [46,47]. In addition to detoxifying ROS, PCs, GSH, and MTs can also ameliorate Cu excess by chelating Cu ions in the cytosol. The Cu-GSH and/Cu-PCs formed can be transported into the vacuole by ABC transporter, thus maintaining cellular redox status [4,48]. Navarrete et al. observed that Cu excess-induced increments in the concentrations of PCs and GSH and the relative levels of transcripts encoding MTs in *Ulva compressa* depended on the Cu concentrations in the culture medium and the Cu exposure time, concluding that they might be involved in the accumulation and detoxification of Cu in a coordinated and complementary manner [49]. Mishra et al. indicated that the antioxidant system and thiol metabolism complemented each other during arsenate detoxification in *Ceratophyllum demersum* [50]. In rice, Mostofa et al. reported that GSH addition mitigated Cu excess-induced increases in O_2_^-^, H_2_O_2_, MDA, GSH, GSSG, and DHA levels, and GR, SOD, APX, and MDHAR activities and decreases in CAT and GST activities, ASC/DHA and GSH/GSSG ratios, and ASC level in leaves, while GSH addition further promoted Cu excess-induced increases in GuPX activity and PCs level in leaves. This suggested that in addition to S metabolism, antioxidant system played a role in the GSH-mediated mitigation of oxidative stress in the leaves of Cu-exposed seedlings [21]. Our findings suggested that B addition lessened the impairments of Cu excess on S metabolism and S-containing compounds in leaves and roots (Figure 3 and Figure 5). A previous study from my group indicated that Cu concentrations in RCu350 and LCu350 were greater at B2.5 than at B25 [7]. Therefore, our results that Cu350 increased the concentrations of PCs, MTs, and TNP-SH in RB2.5 and LB2.5, but not in RB25 and LB25 (Figure 5) agreed with the increased demand for the detoxification of Cu. This also agreed with the findings that Cu excess strongly lowered GSH (a precursor of PCs) level, but greatly increased PC2 level in terrestrial macrolichens [51].

MG can act as both a signal molecule at low level and a cytotoxin at high level. Elevation of MG can induce the production of ROS [15]. MG is mainly degraded by glyoxalases. Gly I catalyzes the conversion of MG to SLG using a GSH molecule. Then the formed SLG is converted into D-lactate acid through Gly II, while producing a GSH molecule [52]. GSH participates in both direct and indirect degradation of MG, and its availability is linked to MG removal [16]. We found that for leaves, MG was positively (negatively) related to Gly II activity (GSH level), and exhibited a downward trend with the increase of Gly I activity (Figure S2A), and that for roots, MG level was negatively correlated with Gly I activity, Gly II activity, and GSH level, respectively (Figure S2B). These results indicated that B addition alleviated MG accumulation in LCu350 and RCu350 by reducing the MG production and keeping a higher capacity to detoxify MG (Figure 1D, Figure 4E and Table 1).

It is pointed out that the coordinated actions of ROS and MG removal systems are necessary for addressing the elevated production of ROS and MG [16]. Since GSH is involved in both ASC-glutathione cycle and MG detoxification system, GSH may serve as an interactive point between antioxidant defense and MG scavenging systems [15]. In addition to reducing, detoxifying, complexing, chelating, and compartmentalizing metals, GSH by itself and its metabolizing enzymes (viz., glyoxalases, GR, DHAR, GST, and glutathione peroxidase) can act additively and coordinately to protect effectively plants against oxidative damage caused by MG and/or ROS [53]. Overexpression of either Gly I or Gly II individually or together enhanced transgenic tobacco plants zinc (Zn) tolerance by sequestering excessive Zn in roots, elevating the level of PCs and the ability to maintain glutathione homeostasis, and subsequently reducing MG and MDA accumulation in transgenic plants during exposure to Zn toxicity [54]. Therefore, the coordinated actions of ROS and MG removal systems might be involved in protecting roots and leaves from oxidative damage caused by Cu excess.

**Figure 7:**
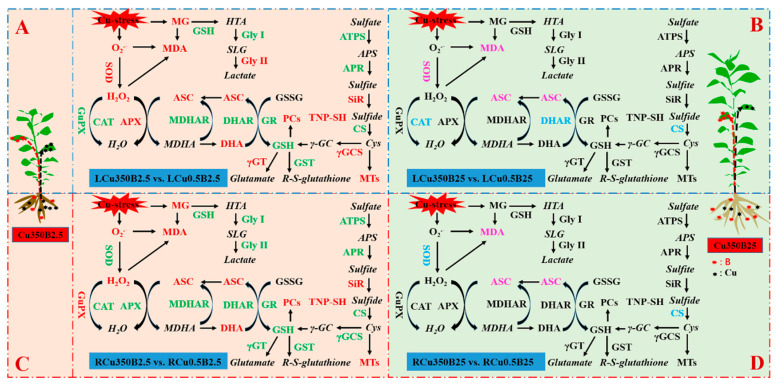
A model for the B-mediated alleviation of oxidative damage caused by Cu excess in *C. sinensis* leaves and roots. Red and pink: upregulation, with a greater change in “red” than in “pink” when comparison between (**A**,**B**) or between (**C**,**D**); Green and blue: downregulation, with a greater change in “green” than in “blue” when comparison between (**A**,**B**) or between (**C**,**D**); Black and plain, unchanged metabolites and enzymes; Black and italics, undetermined metabolites in this study.

Taken together, B addition reduced the Cu excess-stimulated generation and accumulation of ROS and MG and the impairments of Cu excess on ROS (the antioxidant enzymes, ASC-glutathione cycle, and thiol-based antioxidant system) and MG detoxification systems in leaves and roots, thereby protecting them from oxidative damage via the coordinated actions of ROS and MG removal systems (Figure 7). Therefore, the study revealed a novel mechanism of the B-mediated Cu internal detoxification. The alleviating mechanism was a supplement to the previous study that the internal detoxification of Cu by LMWCs played a role in the B-mediated alleviation of Cu toxicity in citrus [8].

### 4.3. The Pathways Involved in the B-Mediated Amelioration of Oxidative Injury Caused by Cu Excess Differed between Leaves and Roots

Our previous studies showed that B supply had a significant impact on Cu distribution in *C. sinensis* roots, stems and leaves [7], and that the responses of the total phenolic concentrations and the related enzyme activities to Cu and B treatments and the responses of ROS and MG biosynthesis and their removal systems to various Cu and pH treatments differed between *C. sinensis* leaves and roots [8,12]. It is known that phenolic compounds can serve as the source of antioxidants and the chelating agents of HMs decreasing the formation of free radicals, thereby protecting plants against oxidative damage [55]. Exogenous application of NO and salicylic acid (SA) reduced Cu excess-induced oxidative damages in rice and tomato leaves and roots. The response of ROS (H_2_O_2_) generation and their detoxification systems to Cu and SA (NO) treatments differed between rice (tomato) leaves and roots [56,57]. Maize, *Arabidopsis*, and grapevine roots and leaves exhibited different characteristics of oxidative stress and antioxidant defense in response to Cu exposure [58,59,60]. Therefore, the pathways involved in the B-induced amelioration of oxidative stress caused by Cu excess might differ between leaves and roots. To test the hypothesis, we analyzed the correlations between 31 leaf and 31 root parameters (Figure S2C). We observed that 6 indexes in the leaves were negatively correlated with the corresponding indexes in the roots, and one leaf parameter displayed a downward trend with the increase of the corresponding root parameter, suggesting that ROS and MG generation and their scavenging systems in the leaves and roots responded differently to Cu and B treatments. Additionally, we analyzed the response patterns of 31 indexes in the leaves and roots to various Cu and B treatments using the PCoA (Figure 6A), and found that huge differences existed in the response patterns of ROS and MG production and their removal systems to Cu and B treatments between the leaves and roots. For instance, Cu350 increased the SAPR, HPR, and the levels of ASC, TA, MTs, PCs, and TNP-SH in RB2.5, RB10, and LB2.5, while it increased the MG concentrations in LB2.5, LB10, and RB2.5 (Figure 1, Figure 4 and Figure 5). Cu350 increased or did not alter the APX, SOD, γGCS, γGT, and Gly II activities, and decreased or did not affect the GuPX activity in the leaves, but the case with the roots was exactly the opposite (Figure 2 and Figure 3 and Table 1).

B2.5 reduced the GuPX activity in LCu350 and the SOD and Gly II activities in RCu350, but did not affect the GuPX activity in RCu350 and the SOD and Gly II activities in LCu350 relative to B25 (Figure 2 and Table 1). B2.5 increased the SiR activity in RCu350, but did not alter its activity in LCu350 relative to B25 (Figure 3). B2.5 increased (decreased) the APX, γGTγ, and GCS activities in LCu350 (RCu350) relative to B25 (Figure 2 and Figure 3).

Taken together, the pathways involved in the B-induced amelioration of oxidative stress caused by Cu excess differed between the leaves and roots (Figure 7).

## 5. Conclusions

Our findings supported the hypothesis that B addition mitigated Cu excess-induced oxidative damage in the leaves and roots by reducing Cu excess-induced formation and accumulation of ROS and MG and by counteracting the impairments of Cu excess on ROS (antioxidant enzymes, ASC-glutathione cycle, and thiol-based antioxidant system) and MG detoxification systems. The alleviating mechanism of B on Cu toxicity in this study was complementary to our previous study [8]. Therefore, this present study provided a novel insight into the mechanism underlying the B-mediated amelioration of oxidative stress caused by Cu excess in plant leaves and roots, and a theoretical basis for developing a new way to mitigate citrus Cu toxicity. However, further studies are needed to clarify the molecular mechanisms which B reduces Cu toxicity in citrus.

## Figures and Tables

**Table 1:** The mean (± SE, *n* = 4) activities of Gly I and Gly II in leaves and roots of *C. sinensis* seedlings under different Cu and B treatments.

Activities	Treatments					
	Cu0.5B2.5	Cu0.5B10	Cu0.5B25	Cu350B2.5	Cu350B10	Cu350B25
Leaves						
Gly I (nmol MG g^−1^ FW s^−1^)	133.9 ± 8.1 a	117.3 ± 6.3 a	125.9 ± 6.6 a	92.6 ± 7.0 b	129.5 ± 12.0 a	120.9 ± 6.2 a
Gly II (nmol SLG g^−1^ FW s^−1^)	17.7 ± 1.8 b	17.5 ± 1.7 b	16.7 ± 1.8 b	23.0 ± 2.0 a	20.0 ± 1.3 ab	18.3 ± 1.5 ab
Roots						
Gly I (nmol MG g^−1^ FW s^−1^)	84.6 ± 4.6 a	82.8 ± 5.8 a	84.4 ± 3.8 a	59.5 ± 7.1 b	80.3 ± 7.9 a	92.0 ± 6.8 a
Gly II (nmol SLG g^−1^ FW s^−1^)	13.3 ± 0.7 a	13.0 ± 1.1 a	12.9 ± 1.1 a	7.7 ± 0.7 b	10.7 ± 0.5 a	11.5 ± 0.9 a

Within the same row, the means followed by the different letters were significantly different according to two-way ANOVA followed by the LSD at *p* < 0.05. The *F* values and the *p* values for Cu, B, and Cu × B were listed in Table S1. Cu0.5B2.5, 0.5 μM Cu + 2.5 μM B; Cu0.5B10, 0.5 μM Cu + 10 μM B; Cu0.5B25, 0.5 μM Cu + 25 μM B; Cu350B2.5, 350 μM Cu + 2.5 μM B; Cu350B10, 350 μM Cu + 10 μM B; Cu350B25, 350 μM Cu + 25 μM B.

## Data Availability

The data presented in this study are available in the article.

## Supplementary Materials

The following supporting information can be downloaded at: https://www.mdpi.com/article/10.3390/antiox13030268/s1, Figure S1: Principle component scatter plot for 62 indexes (31 leaf + 31 root indexes) in leaves and roots of 2.5 (A), 10 (B), and 25 (C) μM B-treated *Citrus sinensis* seedlings at various Cu (0.5 and 350 μM) levels, and in leaves and roots of 0.5 (D) and 350 (E) μM Cu-treated *C. sinensis* seedlings at various B (2.5, 10, and 25 μM) levels; Figure S2: Pearson correlation coefficient matrices for the mean values of 31 physiological parameters in leaves (A), roots (B), and between leaves and roots (C) of *C. sinensis* seedlings; Table S1: All parameters for two-way ANOVA in leaves and roots.
