# Supplementary material for: Boron Reduced Copper Excess-Induced Oxidative Damage in Citrus sinensis by Modulating Reactive Oxygen Species and Methylglyoxal Formation and Their Detoxification Systems
Source: Antioxidants (Basel). 2024 Feb 22;13(3):268. doi: 10.3390/antiox13030268 (PMC10967280; doi:10.3390/antiox13030268)
Supplement: Supplementary file 1 [file antioxidants-13-00268-s001.zip › antioxidants-2882024-supplementary.pdf]

Supplementary Data (Tables S1 and Figures S1-S2)

Table S1. All parameters for two-way ANOVA for in leaves and roots

Parameters	Tissue	Cu		B		Cu × B	
		F	*p*	F	*p*	F	*p*
HPR	Leaves	21.3	0.0002	6.3	0.0084	3.9	0.0393
	Roots	46.5	0.0001	8.6	0.0023	8.0	0.0033
SAPR	Leaves	24.3	0.0001	3.8	0.0423	7.3	0.0046
	Roots	19.8	0.0003	4.3	0.0292	3.5	0.0527
MDA	Leaves	377.6	0.0001	46.2	0.0001	54.9	0.0001
	Roots	415.9	0.0001	63.3	0.0001	62.2	0.0001
MG	Leaves	19.0	0.0004	7.7	0.0038	7.4	0.0046
	Roots	3.7	0.0721	2.2	0.1376	1.1	0.3421
CAT	Leaves	113.6	0.0001	0.9	0.4327	3.9	0.0393
	Roots	45.5	0.0001	10.7	0.0009	9.7	0.0014
APX	Leaves	18.9	0.0004	3.1	0.0702	2.7	0.0974
	Roots	3.6	0.0725	5.2	0.0165	3.3	0.0615
DHAR	Leaves	52.7	0.0001	1.2	0.3253	3.6	0.0480
	Roots	30.1	0.0001	16.4	0.0001	11.6	0.0006
MDHAR	Leaves	8.9	0.0080	2.5	0.1108	1.6	0.2298
	Roots	3.1	0.0932	5.0	0.0188	3.4	0.0575
GR	Leaves	3.8	0.0675	1.7	0.2036	1.8	0.1924
	Roots	7.9	0.0114	4.5	0.0262	6.8	0.0064
GuPX	Leaves	11.6	0.0032	4.6	0.0241	5.741	0.0118
	Roots	9.0	0.0076	0.4	0.6919	1.1	0.3629
SOD	Leaves	37.2	0.0001	1.0	0.3738	1.3	0.3021
	Roots	49.0	0.0001	4.0	0.0370	3.2	0.0651
ATPS	Leaves	5.0	0.0378	1.6	0.2257	2.0	0.1607
	Roots	0.2	0.6564	7.1	0.0054	6.5	0.0076
APR	Leaves	12.1	0.0026	1.2	0.3270	1.0	0.3924
	Roots	3.8	0.0658	2.4	0.1155	2.7	0.0926
SiR	Leaves	16.5	0.0007	0.9	0.4107	0.8	0.4660
	Roots	8.8	0.0082	2.8	0.0899	3.0	0.0739
CS	Leaves	91.5	0.0001	3.6	0.0493	4.7	0.0235
	Roots	95.7	0.0001	5.5	0.0137	7.8	0.0036
γGCS	Leaves	35.4	0.0001	1.8	0.1965	2.4	0.1160
	Roots	13.7	0.0016	3.2	0.0668	2.2	0.1437
γGT	Leaves	24.3	0.0001	7.6	0.0041	5.8	0.0112
	Roots	10.9	0.0040	2.0	0.1586	2.2	0.1356
GST	Leaves	37.4	0.0001	7.3	0.0048	16.0	0.0001
	Roots	8.9	0.0079	1.1	0.3647	2.6	0.0988
Gly I	Leaves	3.1	0.0976	1.1	0.3583	5.9	0.0107
	Roots	1.7	0.2031	3.4	0.0546	3.7	0.0455
Gly II	Leaves	5.1	0.0361	1.4	0.2647	0.6	0.5494
	Roots	18.4	0.0004	2.1	0.1489	3.1	0.0673
TA	Leaves	13.6	0.0017	8.9	0.0021	12.2	0.0005
	Roots	95.4	0.0001	4.7	0.0226	4.3	0.0292
ASC	Leaves	8.7	0.0085	6.8	0.0064	9.8	0.0013

	Roots	62.1	0.0001	2.3	0.1328	2.1	0.1520
DHA	Leaves	8.4	0.0097	2.8	0.0849	3.2	0.0631
	Roots	17.3	0.0006	2.3	0.1241	2.0	0.1688
ASC/TA	Leaves	2.0	0.1761	0.1	0.8768	0.4	0.6615
	Roots	1.1	0.3016	0.5	0.6412	0.4	0.6623
TG	Leaves	3.5	0.0793	1.2	0.3305	1.0	0.3709
	Roots	2.8	0.1097	1.5	0.2467	1.2	0.3313
GSH	Leaves	3.4	0.0824	0.9	0.4111	0.9	0.4370
	Roots	3.0	0.1027	1.6	0.2290	1.3	0.3016
GSSG	Leaves	0.2	0.8966	0.1	0.8840	0.2	0.7828
	Roots	0.0	0.9765	0.0	0.9977	0.0	0.9913
GSH/TG	Leaves	2.6	0.1226	0.3	0.7126	0.2	0.7876
	Roots	2.6	0.1224	0.6	0.5479	0.6	0.5816
MTs	Leaves	13.2	0.0019	3.7	0.0446	3.4	0.0565
	Roots	52.6	0.0001	9.6	0.0015	10.6	0.0009
PCs	Leaves	18.2	0.0005	7.0	0.0057	6.5	0.0076
	Roots	20.0	0.0003	3.5	0.0526	2.7	0.0960
TNP-SH	Leaves	18.0	0.0005	6.9	0.0061	6.3	0.0084
	Roots	19.7	0.0003	3.5	0.0532	2.7	0.0949

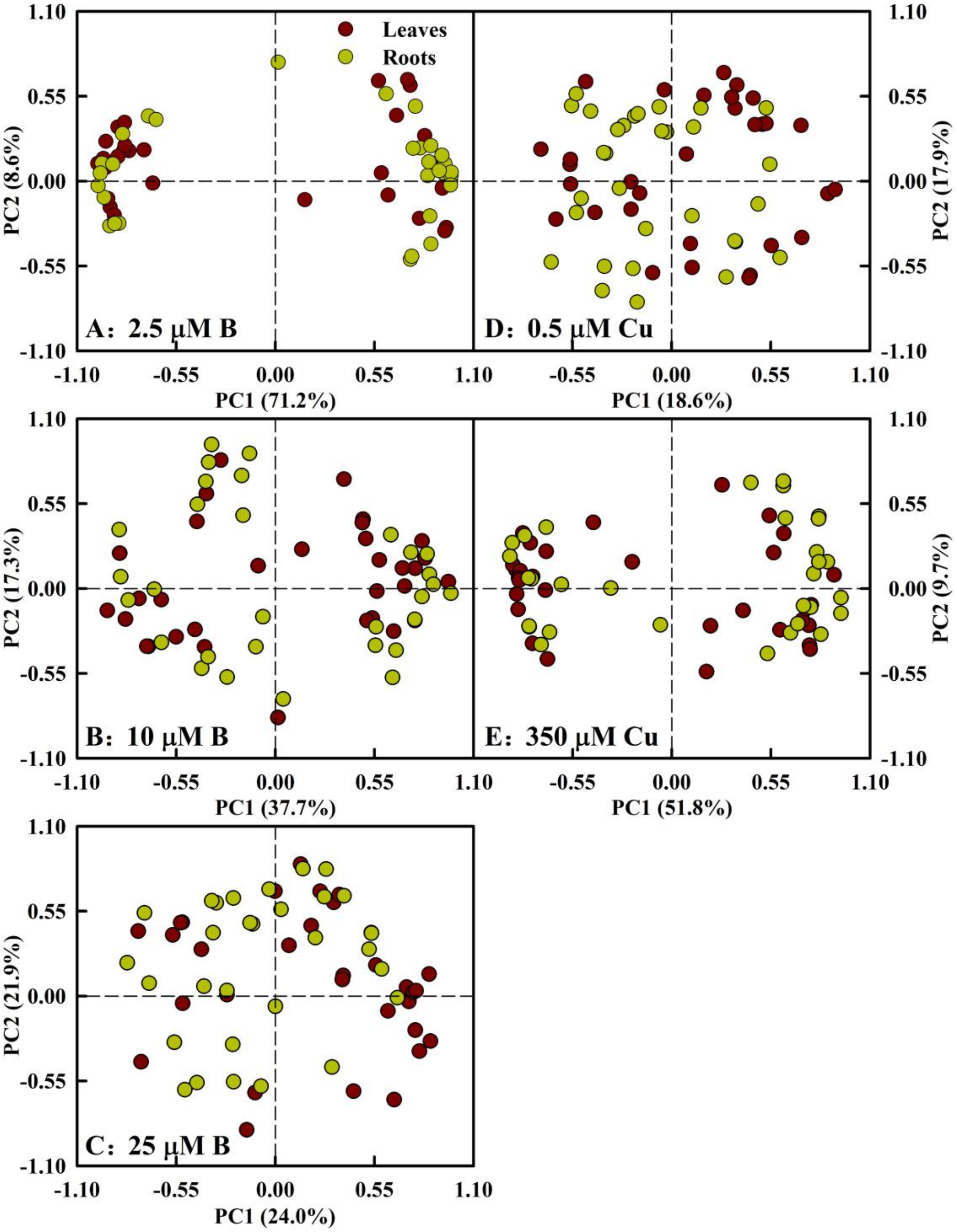

Fig. S1. Principle component scatter plot for 62 indexes (31 leaf + 31 root indexes) in leaves and roots of 2.5 (A), 10 (B), and 25 (C) μM B-treated *Citrus sinensis* seedlings at various Cu (0.5 and 350 μM) levels, and in leaves and roots of 0.5 (D) and 350 (E) μM Cu-treated *C. sinensis* seedlings at various B (2.5, 10, and 25 μM) levels.

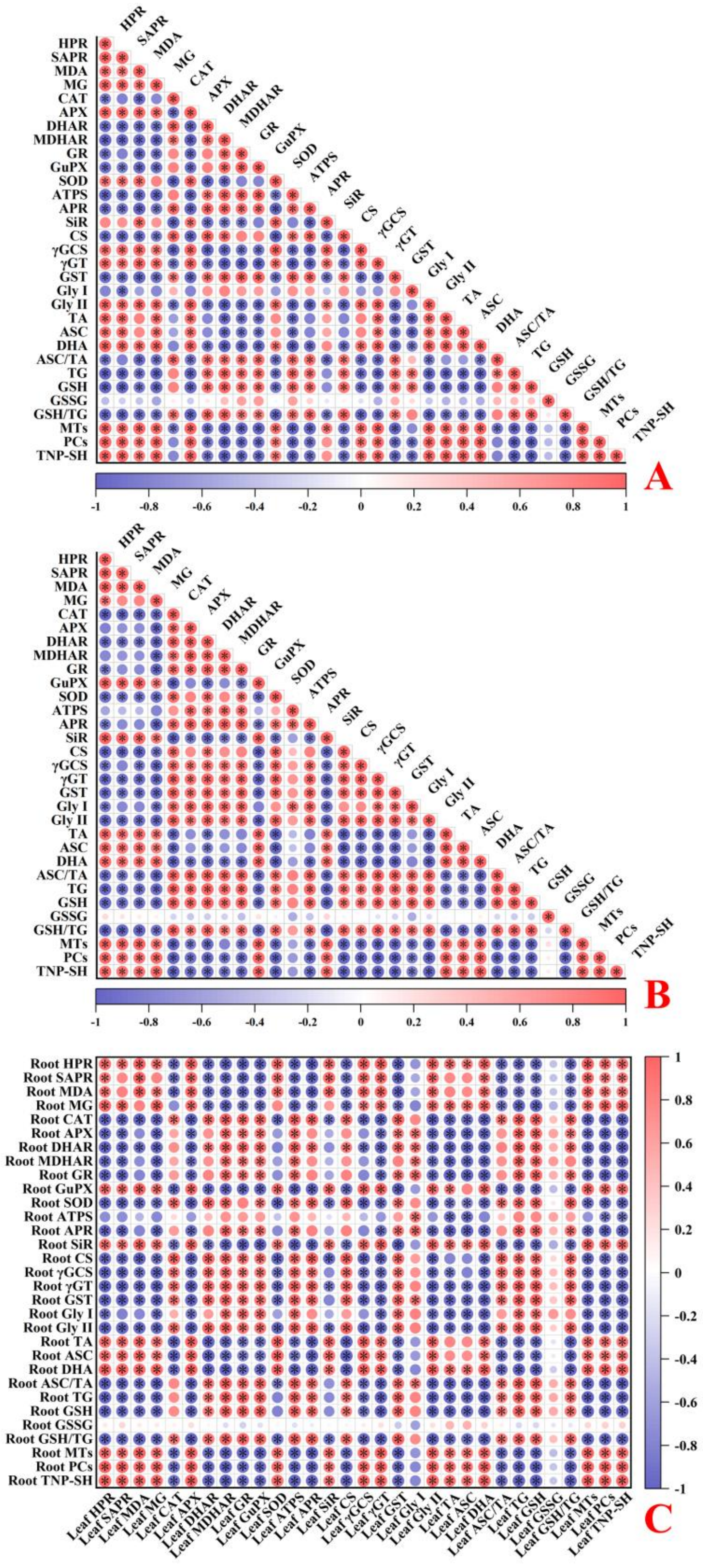

Fig. S2. Pearson correlation coefficient matrices for the mean values of 31 physiological parameters in leaves (A), roots (B), and between leaves and roots (C) of *C. sinensis* seedlings.
